# Predictors of Nursing Faculty Job and Career Satisfaction, Turnover Intentions, and Professional Outlook: A National Survey

**DOI:** 10.3390/healthcare11142099

**Published:** 2023-07-24

**Authors:** Sheila A. Boamah, Michael E. Kalu, Farinaz Havaei, Kimberly McMillan, Emily Belita

**Affiliations:** 1 Faculty of Health Sciences, School of Nursing, McMaster University, 1280 Main Street West, Hamilton, ON L8S 4K1, Canada; belitae@mcmaster.ca; 2 Faculty of Health Sciences, School of Rehabilitation Science, McMaster University, 1400 Main Street West, Institute for Applied Health Science Building, Hamilton, ON L8S 1C7, Canada; kalum@mcmaster.ca; 3 Faculty of Applied Science, School of Nursing, University of British Columbia, T201-2211 Wesbrook Mall, Vancouver, BC V6T 2B5, Canada; havaei@ubc.ca; 4 Faculty of Health Sciences, School of Nursing, University of Ottawa, 451 Smyth Rd, Ottawa, ON K1H 8M5, Canada; kim.mcmillan@uottawa.ca

**Keywords:** nursing faculty, job and career satisfaction, turnover intentions, work–life balance, retention

## Abstract

Background: Retaining talented and experienced nurses in clinical practice and academia is crucial for maintaining continuity, ensuring high-quality care and education, and fostering a positive work environment. Although factors influencing nursing staff retention are well documented, little is known about how workplace factors impact nursing faculty retention outcomes. Methods: A national survey involving 645 nursing faculty across Canada was undertaken. Multivariate regression analysis with interaction effects was conducted to determine the association between work-related factors (i.e., workplace culture and work–life imbalance) and faculty job and career satisfaction, turnover intentions, and professional outlook. Results: Supportive workplace culture positively influenced faculty job and career satisfaction and professional outlook, while it negatively impacted turnover intentions. Conversely, work–life imbalance decreased faculty job and career satisfaction and professional outlook (i.e., confidence in nursing program, profession), and it increased intentions to leave the job. Conclusion: Our results offer insights into the work–life experiences of Canadian faculty members and shed light on key factors that impact their job-related outcomes. In the context of competing resources, every effort must be made to improve modifiable workplace factors such as the academic work environment and create targeted interventions and policies to promote faculty retention.

## 1. Introduction

Recruitment and retention of nurses is an important global concern for healthcare organizations. Prior to the COVID-19 pandemic, the global nursing shortage was estimated at 5.9 million in 2018, much of which was concentrated in low- and middle-income countries (89%), as high-income countries often draw nurses from other nations [1]. The United States Bureau of Labor Statistics (2020) projects that 1.2 million new registered nurses (RNs) will be needed by 2030 to address the current global shortage [2,3]. This projection, however, will lead to increasing demands for academic faculty to educate and train professional nurses to enter the workforce. Currently, there are not enough qualified nursing educators/faculty [4]. The shortage of nursing faculty is compounded in most countries, including Canada, as the healthcare workforce is aging alongside the population. A 2021 scoping review revealed that this shortage is primarily influenced by multifaceted factors, such as a limited number of qualified PhD-prepared nursing faculty available to replenish the aging nursing faculty workforce, employment conditions, organizational support, and personal factors [5]. Research has shown that employment conditions—namely, heavy workload, lack of leadership support, mentorship, collegiality, and defensive/negative department culture—significantly decrease faculty members’ effectiveness, engagement, productivity, job satisfaction, and intention to remain in the job; thus, these are important determinants of retention rates [6,7,8].

Building human capital through the recruitment and retention of faculty is an important strategy for maintaining and expanding the nursing workforce. Although nursing staff retention has been the subject of a vast number of studies, few studies have focused on issues related to the retention of nursing faculty [9,10], and even fewer studies have explored the work–life experiences of Canadian nursing faculty [11,12]. The present study seeks to address this gap by developing a more nuanced understanding of how work–life experiences and organizational factors (i.e., workplace culture and work–life imbalance) influence nursing faculty retention outcomes, such as job and career satisfaction, professional outlook, and turnover intentions/voluntary departure from their current institutions. Understanding the specific factors that influence job-related outcomes among faculty members will allow for targeted interventions and initiatives that can contribute to a more positive and fulfilling work environment. Essentially, this will help in building a stronger and an optimal nursing workforce.

The term “nursing faculty” is used in this paper to refer to nurses who occupy academic roles within a higher education institution, encompassing various positions, such as tenured, tenure-track, non-tenured teaching, and research-oriented streams.

### 1.1. Literature Review

#### 1.1.1. Workplace Culture

In the organizational literature, workplace culture is defined as the shared values, beliefs, behaviors, and practices that characterize an organization and shape how its employees interact with one another and external stakeholders [13]. The workplace culture can exert various impacts, both positive and negative, on employee morale, commitment, productivity, physical wellbeing, emotional health, and the leadership approach adopted by supervisors. Studies have consistently shown that a positive workplace culture (or environment) is strongly associated with retention outcomes such as job satisfaction. For instance, a 2018 systematic review by Arian et al., based on 74 articles, found that factors such as organizational or workplace culture, support, healthy work environment, managerial leadership style, and effective mentorship significantly affect job satisfaction among nurse educators [8]. In a study of academic faculty, Boamah linked positive workplace culture to increased job satisfaction and decreased burnout [14]. Similarly, Xie et al. found that organizations with constructive cultures were more successful in fostering supportiveness and embracing collective values, customs, and social behaviors that promote job satisfaction among clinical/staff nurses [15]. In a healthy workplace, there are family-supportive polices that ensure that employees are empowered and have a strong work–life balance. The culture created by the leadership can determine whether employees experience work–life balance or feel pressured by their superiors, leading to an imbalance.

#### 1.1.2. Work–Life Imbalance

Work–life balance is characterized by an employee’s ability to effectively manage and fulfill the competing demands of work and personal life [16]. Conversely, work–life imbalance refers to an individual’s inability to balance work demands with personal life responsibilities, resulting in physical and emotional exhaustion [16]. Employees often encounter work–life imbalance when the lines between their personal and professional lives become blurred or are inadequately defined by their employer. Several studies have investigated the association between work–life imbalance and job dissatisfaction, increased turnover intentions, and high turnover. In a 2018 study of Dutch nursing faculty, Janssen and colleagues found that work–life imbalance was significantly associated with decreased job satisfaction among faculty [17]. Similarly, a 2023 US study reported a negative association between work–life imbalance and professional quality-of-life outcomes (i.e., average compassion satisfaction/fatigue, increased burnout, and secondary traumatic stress) among nursing faculty [18]. Furthermore, a study by Park et al. found that work–life imbalance significantly predicted intentions to leave among nursing faculty in the Republic of Korea, indicating a potential relationship between work–life imbalance and turnover intentions [19]. These findings suggest that work–life balance is essential for faculty retention and for sustaining a healthy workforce.

#### 1.1.3. Job-Related Retention Outcomes

##### Job Satisfaction

Job satisfaction, defined as how people feel about their job and its aspects [20], is a complex phenomenon with several predictors and mediating factors. Such factors include, but are not limited to, leadership style and characteristics, job design, compensation packages (or salary), working conditions, social relationships, perceived opportunities elsewhere, and levels of aspiration and need achievements [12]. Job satisfaction has been extensively studied among clinical nurses, but sparingly among academic nurses. Lu and colleagues, in a systematic review including 59 articles, reported that the job satisfaction of hospital nurses is related to an empowering work environment, organizational commitment, professional commitment, social capital, patient satisfaction, and the patient–nurse ratio, with several mediating factors [6]. A meta-analysis of 62 studies from 1980–2009 reported 27 job satisfaction predictors, of which task requirements, empowerment, and control were found to have the largest effect sizes on the job satisfaction of frontline registered nurses (RNs) [7]. The study claimed that predictors of nurses’ job satisfaction might be different than previously thought, indicating that some factors may be more important predictors than others, such as professional autonomy, control, and empowerment [7]. Among nursing educators, Arian et al. reviewed the literature on factors affecting job satisfaction in nurse educators, and they included 74 studies and categorized the factors into six levels, including personal, organizational, managerial, academic, professional, and economic [8]. The recurring theme across these systematic reviews, regardless of the population of nurses, was that job satisfaction is an independent indicator of one’s intention to leave or remain in the nursing profession, and the factors influencing job satisfaction change over time depending on the sociopolitical climate.

##### Career Satisfaction

Career satisfaction refers to the subjective perception and evaluation of one’s professional growth and accomplishments, including the contentment with one’s career choice, advancement, and career recognition. Career satisfaction has been intricately linked to both the intrinsic and extrinsic aspects of the career [21]. Although career satisfaction and job satisfaction are often used interchangeably in the literature, they are distinct concepts. While the former term focuses on an individual’s overall affective orientation towards their professional career and is often assessed with questions such as “are you satisfied with your career as a nurse?”, the latter term is assessed with similar questions but is focused on the job roles, such as an educator or care coordinator. Career satisfaction can be measured subjectively by considering the individual’s perceived value, respect, recognition, and sense of community. Career satisfaction is often regarded as a significant indicator of one’s personal career success. Although some studies [22,23] have examined the influence of workplace factors such as mentorship and burnout on career satisfaction, there is limited evidence and understanding on predictors of career satisfaction among the general nursing population, and even fewer studies among nursing faculty. As such, this study aims to identify factors associated with nursing faculty career satisfaction.

##### Intention to Leave the Job

Intention to leave the job (or turnover intention) is defined as when an employee contemplates leaving their organization, which is an important and most consistent predictor of actual turnover [24]. While the intention to leave the job has been studied primarily among other nursing populations (e.g., nurses in clinical settings), studies exploring why nursing faculty leave or stay in their job are limited. In 2011, Gormley and Kennerly sought to understand the factors that contributed to US nursing faculty turnover and found that organizational climate intimacy and disengagement, affective and continuance organizational commitment, and role ambiguity were significant predictors of turnover intention [25]. In a similar design with a sample of 808 US nursing faculty, Candela et al. reported that perceptions of nursing administration’s support and teaching expertise positively predicted faculty members’ intent to stay in their academic organization [26]. Using a photovoice approach, Kirkham sought to understand nurse educators’ lived experiences regarding the quality of their work environment and its link to nurse retention [27]. The author found that nursing educators’ experiences of professional autonomy and control over their work influenced their decision to stay in or leave their organization. This finding highlights the impact of workplace culture on nurse retention. In sum, the intention to leave the job is primarily influenced by job and career satisfaction and, to an extent, professional outlook, with a plethora of factors such as workplace culture and public health events (e.g., COVID-19) mediating these relationships.

##### Professional Outlook

Professional (or career) outlook refers to an individual’s attitude towards their profession, which typically involves the expected changes in that profession [28]. Given the current nursing workforce shortages and the recent pandemic, it is important to understand how these ongoing challenges influence nursing faculty’s professional outlook and their confidence in the current direction of the nursing profession, programs, and the health workforce [12]. In many regards, the COVID-19 pandemic exposed gaps and opportunities in nursing education. During the peak of the pandemic, many nursing programs halted on-site course delivery and began redesigning program delivery to ensure that nursing education standards were not compromised. With limited time to transition from face-to-face classes and clinical placement to an online format, including navigating remote teaching, faculty members faced many challenges that may have influenced their professional outlook [29]. Studies exploring the impact of the COVID-19 pandemic on nursing education do exist [30,31], but very few have assessed faculty members’ professional outlook, despite the devastating impact of the pandemic on the nursing workforce. The COVID-19 pandemic, which can be seen as a source of negative career shocks leading faculty nurses to reassess their values and goals in nursing, provides an opportunity to investigate faculty members’ perspectives on nursing programs, institutions, and the profession. Such an investigation can offer insights into ways to better support current and prospective faculty and inform workforce strategies.

Based on the above discussion, this study aims to investigate the extent to which workplace culture and work–life imbalance impact job-related retention outcomes such as job and career satisfaction, intention to leave the job, and professional outlook among nursing faculty, as well as the interaction effects of these factors.

## 2. Materials and Methods

### 2.1. Study Design

This study used a descriptive cross-sectional survey design—a component of a two-phase sequential explanatory mixed-methods study published elsewhere [24]. This study was approved by the Hamilton Integrated Research Ethics Board (HiREB-#1477). The participants were provided a cover letter that explained the objectives of the study and, upon providing informed consent, they were allowed to participate. 

### 2.2. Participants and Sampling

The target population included nursing faculty members working in Canadian colleges and universities, in either a full-time or part-time research- or teaching-track position. Exclusion criteria consisted of adjunct faculty and visiting professors. Convenience sampling was used to recruit participants. Eligible faculty members were identified through their online profiles for their respective universities or college affiliations. The participants were sent an email invitation, which included the description and purpose of the study, inclusion criteria, and a link to a structured questionnaire hosted on the Qualtrics online survey platform. The survey contained a letter explaining the potential risks of the study, the benefits of completing the survey, and strategies to ensure the respondents’ anonymity (see study protocol for additional information [24]). We estimated that the total sample size of 645 was sufficient to have 80% power for the overall regression equation (e.g., adjusted R-squared of 0.017, as found in a past study [24]) in our study, assuming a confidence interval of 95% with a 5% margin of error.

### 2.3. Data Collection

Data were collected between May and July 2021. The participants were invited to complete the online survey. The survey included a demographic section that assessed the participants’ age, gender, education level, academic rank, and tenure status, along with other reliable and validated questionnaires assessing the participants’ job and career satisfaction, intention to leave their job, professional outlook, and situational factors such as workplace culture and work–life imbalance. Details of these questionnaires are presented in the subsequent section. A modified Dillman approach was used to increase the response rate [32]. Participants who had not responded to the survey after the initial invitation were sent two subsequent email reminders in weeks 3 and 4. Completion and submission of the survey implied the respondent’s consent to participate in the study.

### 2.4. Outcome Variables 

The outcome variables are job satisfaction, career satisfaction, intention to leave the job, and professional outlook. 

#### 2.4.1. Job Satisfaction

Nursing faculty completed the Global Job Satisfaction Questionnaire (GJS) adapted from the Job Diagnostic Survey [33]. This questionnaire has four questions about the extent to which participants agree or disagree with statements relating to their satisfaction with the job, their coworkers’ satisfaction with the job, their desire to stay in the job until retirement, and the supportiveness of the working environment. Participants were asked to rate each statement on a 5-point Likert scale from 1 (strongly disagree) to 5 (strongly agree), with higher sum scores indicating greater levels of job satisfaction. The GJS has shown acceptable internal consistency and reliability, ranging from 0.78 [34] to 0.88 among nursing populations [12].

#### 2.4.2. Career Satisfaction

Nursing faculty completed a five-item career satisfaction scale developed by Greenhaus et al. [16]. The participants rated each item (e.g., All in all, I am satisfied with my career in nursing) using a 5-point Likert scale ranging from 1 (strongly disagree) to 5 (strongly agree), with higher sum scores indicating greater levels of career satisfaction. This scale has been validated among the nursing population, with an acceptable internal consistency (Cronbach’s alpha = 0.79) [12].

#### 2.4.3. Intention to Leave the Job

Intention to leave the job was measured using Kelloway et al.’s three-item questionnaire [35]: “I plan to leave the job within the next year”, “I have been actively looking for other jobs”, and “I want to remain in my job”. Participants rated the following items using a 5-point Likert scale ranging from 1 (strongly disagree) to 5 (strongly agree). The question “I want to remain in my job” was reverse-coded. A higher sum score indicated greater intent to leave the job. This scale is a well-known, reliable, and validated tool among clinical nurses, frontline nurses [36], and faculty nurses [24], with an acceptable Cronbach’s alpha ranging from 0.80 to 0.92 [36].

#### 2.4.4. Professional Outlook

Professional outlook was assessed using three items that explore how confident nursing faculty are with the current direction of (a) the nursing program they teach in, (b) the college or university they work in, and (c) the overall profession of nursing. The participants rated these items using a 5-point Likert scale from 1 (strongly disagree) to 5 (strongly agree). Each item was treated as the participants’ professional outlook on the nursing program, the college or university, or the nursing profession, and higher sum scores reflected a more positive outlook. In evaluating this construct, face and content validity were tested as essential parts of the initial assessment process with a small of group faculty members (i.e., comprehensibility of instructions and language used in the assessment tool, the response process and relations among variables, as the evidence of validity). In this study, the Cronbach’s alpha was 0.78.

### 2.5. Predictor Variables

The independent variables are work-related factors that could predict the outcome variables described above. These variables included a researcher-developed six-item workplace culture questionnaire, and a validated measure of work–life balance.

#### 2.5.1. Workplace Culture

The faculty members were asked six questions related to aspects of their academic environment, including role expectations and the promotion process. Participants used a 5-point Likert scale from 1 (strongly disagree) to 5 (strongly agree) to rate statements pertaining to workplace culture (e.g., I have a clear understanding of the expectations of my role as it relates to teaching, research, and/or service). These items were tested for face and content validity with a small sample of nursing faculty from three Canadian provinces at different career stages (e.g., assistant, associate, and full professor) to ensure that the statements appropriately and adequately capture the intended construct. We tested the internal consistency of the item, and it was within a good range (Cronbach’s alpha = 0.85) [14].

#### 2.5.2. Work–Life Imbalance

Work-life imbalance was assessed using the modified version of the work interference with personal life (WIPL) scale [37]. The WIPL 7-item questionnaire captures how an employee’s working life affects their ability to maintain a work–life balance. Respondents rated items using a 7-point Likert scale ranging from 1 (not at all) to 7 (all the time). Samples of items include: “my personal life suffers because of work”, and “my job makes personal life difficult”. The total scoring of the WIPL questionnaire ranged from 7 to 49, with lower scores indicating a better work–life balance and high scores representing work–life imbalance. This scale has undergone robust testing for psychometric properties, including construct validity (confirmatory factor analysis: X^2^ = 247, df = 122, CFI = 0.97, RMSEA = 0.06) and internal consistency (Cronbach’s alpha of 0.92) among nursing samples [36].

### 2.6. Data Analysis

Data analyses were conducted using STATA/IC (v14), with the *p*-value for significance set at <0.05. Descriptive statistics were calculated for all sociodemographic characteristics, including measures of central tendency (mean) or dispersion (standard deviation), frequency, and percentages. No data were missing for any of the variables included in this analysis. All continuous data were initially checked to ensure that assumptions were met. We tested for homogeneity of regression variance using the Cook–Weisberg test for heteroskedasticity, for normality of the residuals using the Shapiro–Walk (Calc W) test and visual inspection of the histogram, and for collinearity using the variance inflation factor. All normality assumptions were made. We performed multiple correlation analyses (Spearman’s rank correlation coefficients) to explore the relationships between dependent variables (career and job satisfaction; intention to leave; professional outlook on nursing program, institution, and profession) and independent variables (work–life imbalance and workplace culture). Afterward, six regression analyses were performed to determine the associations between the independent variables and four dependent variables. For each analysis, all independent variables and their interactions—for example, work–life imbalance and workplace culture—were included in the model, and those that were not significant were removed from the model. Using a backward elimination approach, the model with the best improved adjusted R-squared was taken as the final model.

## 3. Results

### 3.1. Demographic Information

We sent emails to 1649 eligible participants, and 645 responded, resulting in a 39.1% response rate. Slightly over half of the respondents (*n* = 336) were 49 years old or younger. Approximately 94% (*n* = 604) identified as female, and 34.1% of participants had a PhD. The majority of the participants (81.2%, *n* = 524) were faculty members at universities, and the remainder worked at colleges (18.76%, *n* = 121). Details of the respondents’ demographic characteristics are shown in Table 1.

### 3.2. Descriptive Statistics

Nursing faculty members were highly satisfied with their career, moderately satisfied with their job, had low intention to leave, and had a moderate outlook on the nursing profession, the nursing program, and their institution. See Table 2 for correlations, means, and standard deviations for the key study variables.

### 3.3. Regression Results 

#### 3.3.1. Factors Associated with Job Satisfaction

A significant regression equation was found (F (2, 642) = 243.03, *p* = 0.001), with an R^2^ of 0.4309. Work–life imbalance (β = −0.20, *p* < 0.001) and workplace culture (β = 0.56, *p* < 0.001) were negatively and positively associated with job satisfaction, respectively (see Table 3). 

#### 3.3.2. Factors Associated with Career Satisfaction

A significant regression equation was found (F (2, 642) = 103.89, *p* = 0.001), with an R^2^ of 0.2445. Work–life imbalance (β = −0.20, *p* < 0.001) and workplace culture (β = 0.39, *p* < 0.001) were negatively and positively associated with career satisfaction, respectively (see Table 3). 

#### 3.3.3. Factors Associated with Intention to Leave the Job

A significant regression equation was found (F (2, 642) = 85.05, *p* = 0.001), with an R^2^ of 0.2095. Work–life imbalance (β = 0.16, *p* < 0.013) was positively associated with intention to leave the job, while workplace culture (β = −0.38, *p* < 0.001) was negatively associated with intention to leave the job (see Table 3).

#### 3.3.4. Factors Associated with Professional Outlook

A significant regression equation was found for confidence in the nursing program (F (2, 642) = 118.71, *p* = 0.001), with and R^2^ of 0.2700; for confidence in the nursing profession (F (2, 642) = 28.36, *p* = 0.001), with an R^2^ of 0.0812; and for confidence in the college/university (F (2, 642) = 92.73, *p* = 0.001), with an R^2^ of 0.2241. Workplace culture was significantly associated with confidence in the nursing program (β = 0.52, *p* < 0.001) and confidence in the college or university (β = 0.44, *p* < 0.001) (see Table 4). Workplace culture (β = 0.26, *p* < 0.001) and work–life imbalance (β = −0.07, *p* = 0.036) were significantly associated with confidence in the nursing profession, but in opposite directions.

All interaction terms were not significant, implying that the effect of a change in the value of an independent variable (e.g., workplace culture) on the mean outcome (e.g., career satisfaction) does not depend on the value of another independent variable (e.g., work–life imbalance).

## 4. Discussion

With the uncertainty associated with the COVID-19 pandemic, our study aimed to investigate how workplace factors influence nursing faculty retention. Among the factors studied, workplace culture was the most significant predictor of all of the job-related retention outcomes. As expected, a supportive workplace culture was associated with higher levels of job and career satisfaction, lower turnover intentions, and a positive professional outlook (i.e., confidence in the nursing program, academic institution, and/or profession). Unsurprisingly, faculty members who experienced work–life imbalance reported lower career and job satisfaction and professional outlook, and were more likely to leave their job than those with perceived work–life balance. The findings of this study add to the existing literature on key factors influencing nursing faculty retention and/or attrition [8,24,26]. The results provide insight that could guide national policies and strategies to improve the academic work environment and subsequent retention efforts amid the national nursing workforce shortages.

The evidence generated from this study reaffirms the findings of earlier research that emphasized the importance of workplace culture [8,12,23]. Regardless of the context or setting, a supportive workplace culture has a profound impact on the effectiveness and efficiency of an organization in mitigating negative outcomes associated with work. The academic culture prior to the pandemic was described as stressful, performance-driven, individualistic, and highly competitive, with an endemic culture of presenteeism, contributing to work–life imbalance, burnout, and job dissatisfaction [38]. Evidently, the pandemic has intensified the stressors in the academic environment and worsened existing systemic barriers, which may further exacerbate the nursing workforce shortage crisis. While some faculty members may have benefited from the pandemic-related opportunities (i.e., new funding and collaborations), others have been severely disadvantaged, including female, early-career, and Black, Indigenous, and people of color (BIPOC) faculty [39]. In our study, nursing faculty members (93.6% self-identified as female) reported a pronounced conflict between work and personal life. Work–life imbalance has been linked to increased burnout and lower job satisfaction, which are significant predictors of employee turnover [12].

Improving the workplace culture is crucial to promoting faculty retention. A negative or defensive workplace culture is a precursor of work–life imbalance, especially if the work environment lacks support and resources [12]. Hence, in this study, we assessed whether interactions between these two factors could influence retention outcomes; however, the interaction effects were not significant. Given that this study was conducted during the COVID-19 pandemic, it is plausible that the pandemic implicitly impacted the observed associations. The cumulative impact of COVID-19 on both academic and clinical nurses’ mental health has led to some nurses quitting their jobs, thereby increasing the workload for the remaining nurses. This invariably causes burnout, leading to further attrition, forming a vicious cycle that impacts retention in an already under-resourced sector [40].

In congruence with previous studies [25,26,27], nursing faculty members in this study reported some intention to leave their job. Although turnover is expected in any industry, high faculty turnover can disrupt academic programs, compromise student learning experiences, and increase recruitment and training costs. Turnover in nursing is well documented in the literature, yet no concrete nationwide strategies have been proposed to curb the nursing faculty shortage in Canada. Numerous studies on the Canadian nursing workforce point to the need for comprehensive, evidence-based strategies and policies to address the issues in the work environment and improve retention [5,11,34]. During the 74th World Health Assembly, the International Council of Nurses proposed strategies including the Health Education and Retraining Opportunity (HERO) funds to increase the capacity of the education sector to train more nurses and reduce the rates of attrition from nursing schools [40]. The HERO funds are intended to support the development and implementation of nursing faculty training programs, such as new teaching methodologies, curriculum design, and assessment. These funds can be used to support nursing faculty in attending conferences and enabling them to be updated with the latest developments in nursing education in the context of the emerging world of artificial intelligence. The HERO funds also include financial incentives such as salary increases, bonuses, loan repayment programs, and scholarships for nurses to pursue higher education or training, enabling them to take on a faculty appointment—either full, part-time, or as clinical instructors.

In this study, nursing faculty had a moderate professional outlook, which refers to an individual’s attitude towards their profession and its expected changes. This finding was congruent with the results of an Australian study, where Takase et al. [41] investigated clinical nurses’ self-image, perceived public image, leadership, caring attitudes, and intentions to leave their job, finding that having a positive self-image regarding aptitude for leadership led to lower turnover intentions. Consistent with the existing literature [42], we found that workplace culture significantly influenced nursing faculty members’ professional outlook. In a descriptive exploratory qualitative study, Emeghebo reported that nurses’ perceptions of their profession are influenced by their work environment and interactions with others [42], and that support from senior colleagues had a significant impact on younger nurses’ professional outlook. In alignment with our results, it is plausible that senior nursing faculty members’ attitudes toward younger educators could provide additional information on faculty retention and stimulate interest among clinical nurses in pursuing careers in academia. Lastly, contrary to our expectations, work–life imbalance was not a significant predictor of poor professional outlook; thus, future research should draw on qualitative methods to shed light on the reasons for this finding.

### 4.1. Study Implications

The findings of this study indicate an urgent need for institutional leaders to address systemic issues that contribute to an unsupportive workplace culture, increased stress, high turnover, and suboptimal mental health among faculty members. Given the current human resources crisis in healthcare, every effort must be made to improve nursing faculty’s satisfaction and retention. Satisfied faculty members are likely to provide high-quality instruction and preparation of future nurses, create a positive learning environment, and offer effective support and guidance to students, leading to improved student performance, retention, and success [43]. Such faculty strive to be more productive in their research endeavors, leading to increased scholarly contributions, publications, and grant acquisition. This, in turn, enhances the reputation of the institution and contributes to advancements in knowledge and innovation.

Based on our findings, academic leaders can implement several innovative strategies to improve nursing faculty retention. Specifically, institutions must recognize the importance of work–life balance by implementing flexible work arrangements and workload management policies. This could include options for remote work, flexible scheduling, teaching assistant support, and reduced teaching loads [13]. Such arrangements can help faculty members to manage their personal and professional responsibilities, reduce stress, and improve job satisfaction. Further, implementing other supportive workplace strategies and programs, such as mentoring or coaching, can be beneficial—especially for new and early-career faculty. Such programs can facilitate career development, assist faculty with navigating the tenure process, and provide a network for professional connections. Creating support systems for faculty, such as counseling services and wellness programs, can contribute to their overall wellbeing, and job and career satisfaction.

A consistent strategy that has been proposed in the organizational and healthcare literature is workload management. If not already in practice, academic institutions should work towards creating standardized workload formulae to use across all departments, regardless of the discipline. For example, there should be consistency in the number of courses assigned (or course release) to every new or tenure-track faculty member at the same institution, if the criteria for the tenure and promotion process are to remain the same. Leaders must create workload policies that are rooted in the principles of equity, transparency, reasonableness, safety, and faculty satisfaction. Inequitable practices create more workload and unequal promotion opportunities for faculty members in certain departments, while advantaging others. In academia, there is an assumption that nursing faculty workload is not equitable with faculty workload in other academic disciplines, as the measures failure to account for various aspects, including clinical practice, maintaining clinical competence, licensure or certification, and other non-classroom activities [44]. Research efforts should prioritize the development of theoretical and operational definitions of faculty workload that encompass key aspects such as clinical practice, research, and service [44]. These definitions should be informed by universities’ promotion and tenure guidelines to ensure comprehensive and equitable assessments of nursing faculty workload. Further, evidence suggests that female academics and BIPOC faculty are often pressured to take on more service and committee work, and this added burden can have detrimental effects on their productivity and satisfaction [39]. Therefore, careful consideration must be applied when assigning workloads.

As our findings indicate, workplace culture plays a crucial role in faculty retention; thus, institutions should foster a collaborative, nurturing, and constructive workplace environment that recognizes the value of all faculty members, regardless of their rank. Academic leaders must pay particular attention to various aspects within the work environment, including conditions, favoritism, language usage, unethical practices, organizational politics, and gender distinctions. Senior leadership should devise employee-friendly and equitable policies that recognize the unequal impact of stressors like COVID-19 on faculty, especially those from underrepresented or marginalized groups [39].

Providing dedicated research time and reduced teaching loads for faculty members engaged in research can significantly enhance their productivity, satisfaction, and contributions to knowledge. Hiring additional support staff, such as administrative assistants or research coordinators, can help alleviate administrative burdens and allow faculty to focus more on teaching and research, which in turn, will reduce burnout and enhance job satisfaction [14]. Offering opportunities for faculty to explore and adopt innovative teaching methods, technologies, and pedagogical approaches can be an extremely effective way of supporting faculty. This can include providing access to instructional designers, technology specialists, and educational resources to facilitate the integration of technology into teaching practices. Academic leaders should offer faculty members opportunities for professional development and career advancement, including funding for conferences, workshops, and training programs, as well as support for research projects and collaborations [45]. 

While external funding is desirable, academic institutions should offer research support including funding for team-based projects, research space and equipment, and assistance with grant applications [45]. Other known and effective strategies include the establishment of interdisciplinary research centers or institutes that promote collaboration across different disciplines. This fosters a sense of community, expands research opportunities, and enhances faculty engagement and job satisfaction. Meaningfully involving faculty members in institutional decision-making processes and providing opportunities for their input helps faculty to feel valued, engaged, and invested in the institution’s success. Further, it is important that faculty members are formally recognized and rewarded for their efforts and achievements at both the departmental and institutional levels. This can include acknowledging exceptional teaching, research, and service through awards, public recognition, and financial incentives, to demonstrate appreciation and create a positive work culture. By implementing these strategies, institutions can create a supportive and engaging environment that values faculty members’ contributions, fosters professional growth, and promotes job satisfaction and retention.

### 4.2. Study Strengths and Limitations

This is among the few studies that have examined the work–life experiences of nursing faculty in Canada. This study advances the current state of knowledge by explaining the relationships between workplace factors and retention outcomes. To the best of our knowledge, this is the first study to assess faculty members’ professional outlook in the context of the COVID-19 pandemic. Although the knowledge generated is insightful, the study’s predictors were assessed only on the basis of a cross-sectional design. In future studies, researchers should consider using longitudinal designs and validating the professional outlook scale. Studies should also explore the effects of other key factors, such as workload, nursing faculty characteristics (e.g., gender), and current position and rank (tenured vs. non-tenure or clinical track vs. teaching track), all of which may improve retention and serve as a strategy to prevent turnover. More research is needed to assess the similarities and differences between groups of nursing educators (college vs. university professors) in various settings and with other practice-based disciplines in academia.

## 5. Conclusions

This study provides practical and valuable evidence of the factors that attract and retain nursing faculty in the ever-changing, competitive environment of academia. In order to curb the global nursing workforce shortages, every effort must be made to improve the quality of the work environment and workplace culture in nursing programs, including creating work–life balance polices and reducing the additional pandemic-related pressures and demands on faculty. Understanding the factors that influence faculty’s satisfaction and retention is essential for succession planning, improving student outcomes, enhancing productivity and research output, promoting collaboration and teamwork, cultivating a positive organizational climate, attracting talent, and supporting faculty wellbeing. This ultimately contributes to the overall success and reputation of academic institutions and, more urgently, to the preparation and retention of the health workforce.

## Figures and Tables

**Table 1:** Demographic characteristics of the respondents (*n* = 645).

Demographic Variable	N	%
Sex		
Female	604	93.6
Male	36	5.6
Other	5	0.8
Age		
≤39 years	145	22.5
40–49 years	191	29.6
50–59 years	195	30.2
≥60 years	106	16.4
Prefer not to say	8	1.2
Highest education		
PhD	220	34.1
Master’s	340	52.7
Bachelor’s	79	12.3
Diploma	6	0.9
Academic rank		
Lecturer	82	12.7
Assistant professor	144	22.3
Associate professor	230	35.7
Full professor	88	13.6
Clinical/sessional instructor	101	15.7
Tenure status		
Tenured	152	23.6
Tenure track	84	13.0
Teaching track	168	26.1
Non-tenure track	149	23.1
Clinical track	92	14.3
Employment status		
Full-time permanent	453	70.2
Full-time temporary	75	11.6
Part-time	117	18.1
Years worked at current organization		
≤1 year	45	7.0
2–5 years	200	31.0
6–10 years	136	21.1
≥10 years	264	40.9
Hours worked per week		
≤35 h	86	13.3
36–40 h	121	18.8
40–45 h	119	18.4
≥46 h	319	49.5
Institution type		
University	524	81.2
College	121	18.8
Institution size		
Small	185	28.7
Mid-size	215	33.3
Large	245	38.0

**Table 2:** Correlations, means, standard deviations, and ranges of the major study variables.

Study Variable	Mean (SD)	Range	1	2	3	4	5	6	7	8
Dependent variables (outcomes)										
1. Career satisfaction	4.08 (0.76)	1–5	-	0.59 **	−0.49 **	0.49 **	0.45 **	0.43 **	−0.33 **	0.46 **
2. Job satisfaction	3.15 (0.99)	1–5		-	−0.56 **	0.56 **	0.50 **	0.37 **	−0.38 **	0.63 **
3. Intent to leave	2.17 (1.02)	1–5			-	−0.44 **	−0.46 **	−0.31 **	0.28 **	−0.43 **
4. Outlook on program	3.47 (1.18)	1–5				-	0.64 **	0.49 **	−0.17 **	0.52 **
5. Outlook on institution	3.49 (1.11)	1–5					-	0.49 **	−0.22 **	0.47 **
6. Outlook on profession	3.58 (1.09)	1–5						-	−0.15 **	0.28 **
Independent variables (predictors)										
7. Work–life imbalance	4.59 (1.38)	1–7							-	−0.33 **
8. Workplace culture	3.37 (0.74)	1–5								-

*Note*: ** = significant *p* ≤ 0.001.

**Table 3:** Multiple regression: the predictors of job satisfaction, career satisfaction, and intention to leave the job.

Predictors	B	95% LCI	95% UCI	T	*p*-Value	β
Job satisfaction						
Workplace culture	0.7535	0.6709	0.8362	17.90	0.001	0.5636
Work–life imbalance	−0.1428	−0.1870	−0.0986	−6.34	0.001	−0.1997
_cons	1.2640	0.8653	1.6628	6.22	0.001	
Career satisfaction						
Workplace culture	0.4006	0.3273	0.4739	10.73	0.001 *	0.3893
Work–life imbalance	−0.112	−0.1510	−0.0726	−5.61	0.001 *	−0.2033
_cons	3.2465	2.8929	3.6001	18.03	0.001	
Intention to leave the job						
Workplace culture	−0.5222	−0.6224	−0.4219	−10.23	0.001 *	0.3894
Work–life imbalance	0.1180	0.0644	0.1717	4.32	0.001 *	−0.2034
_cons	2.9289	2.3703	3.4876	10.3	0.001	

*Note*: LCI: lower confidence interval, UCI: upper confidence interval, B: unstandardized beta, β: standardized beta, * = *p*-values < 0.05 are significant, T = t-value.

**Table 4:** Multiple regression: the predictors of professional outlook.

Predictors	B	95% LCI	95% UCI	T	*p*-Value	β
Nursing program						
Workplace culture	0.8273	0.7154	0.9393	14.51	0.001 *	0.5176
Work–life imbalance	−0.0051	−0.065	0.0548	−0.17	0.868	−0.0059
_cons	0.70516	0.1653	1.2451	2.56	0.011	
Nursing professor						
Workplace culture	0.3787	0.2629	0.4945	6.42	0.001 *	0.2569
Work–life imbalance	−0.0513	−0.1132	0.0106	−1.63	0.104	−0.0651
_cons	2.5349	1.9764	3.0933	8.91	0.001	
University/college						
Workplace culture	0.6623	0.5543	0.7703	12.04	0.001 *	0.4427
Work–life imbalance	−0.0617	−0.1194	−0.0039	−2.10	0.036	−0.0772
_cons	1.5417	1.0207	2.0627	5.81	0.001	

*Note*: LCI: lower confidence interval, UCI: upper confidence interval, B: unstandardized beta, β: standardized beta, * = *p*-values < 0.05 are significant, T = t-value.

## Data Availability

The original contributions presented in this study are included in the article; further inquiries can be directed to the corresponding author.
